# The bleeding diathesis in patients with hereditary haemorrhagic telangiectasia is not due to impaired platelet function

**DOI:** 10.1186/s13023-023-02968-z

**Published:** 2023-11-14

**Authors:** Anne Lørup Lyster, Signe Hedengran Biørn, Anette Drøhse Kjeldsen, Christian Nielsen, Bibi Lange, Annette Dam Fialla, Pernille Just Vinholt

**Affiliations:** 1 Department of Clinical Biochemistry, Odense University Hospital, Winsløws Vej 4, 5000 Odense C, Denmark; 2 Department of Clinical Research, University of Southern Denmark (SDU), Campusvej 55, 5230 Odense M, Denmark; 3 Department of Gastroenterology, Odense University Hospital, J.B. Winsløws Vej 4, 5000 Odense C, Denmark; 4 Department of Oto-Rhino-Laryngology, Odense University Hospital, J.B. Winsløws Vej 4, 5000 Odense C, Denmark; 5 Department of Clinical Immunology, Odense University Hospital, J.B. Winsløws Vej 4, 5000 Odense C, Denmark; 6 Member of VASCERN (The European Reference Network of Rare Multisystemic Vascular Diseases, Paris), The HHT-Centre at Odense University Hospital, 5000 Odense C, Denmark; 7 OPEN, Patient Data Explorative Network, J.B. Winsløws Vej 9A, Third Floor, 5000 Odense C, Denmark

**Keywords:** Hereditary haemorrhagic telangiectasia, Platelet function, Bleeding, Primary haemostasis

## Abstract

**Background:**

Patients with the rare disease; Hereditary haemorrhagic telangiectasia (HHT) often bleed from telangiectatic lesions in mucosal surfaces. Studies suggest that impaired platelet function may also play a role in their bleeding tendency. The aim of the present study was to investigate whether HHT-patients with epistaxis have impaired platelet function.

**Method:**

We conducted a case–control study based on a sample size calculation and included 22 HHT-patients (inclusion criteria: epistaxis severity score ≥ 4, no intake of medicine affecting platelet function the last 5 days, HHT-type 1 or 2, age ≥ 18 years) and 20 controls. We assessed the platelet function with standard haemostasis parameters, flow cytometry (platelet function and micro aggregation), rotational thromboelastometry and Platelet Function Analyzer 200.

**Results:**

We found no significant difference in mean platelet volume and immature platelet fraction and no difference in platelet activation as measured by exposure of CD62P, CD63P and PAC1 binding. Nor did we find a significant difference in platelet aggregation response in HHT-patients compared with the control group for all agonists (thrombin receptor activating peptide, adenosine diphosphate and collagen-related peptide). The PFA-200 analysis was without difference between the two groups and thromboelastometry showed no impairment of global haemostasis.

**Conclusion:**

Reduced platelet function is unlikely to contribute to the frequent and long bleeding episodes that HHT-patients suffer from. We propose that further studies should focus on whether patients with HHT have hypercoagulability.

## Introduction

Hereditary haemorrhagic telangiectasia (HHT) is a rare disease that affects 1 in 6400 persons [1]. The disease is autosomal dominantly inherited, affects angiogenesis and leads to formation of multiple arteriovenous malformations (AVMs) in the lungs, brain and liver as well as formation of telangiectatic lesions in mucosal membranes [1]. The patients experience bleeding from telangiectatic lesions and may also develop arteriovenous malformations. If these malformations are pulmonary, they may cause paradoxical thromboembolic events [2]. Bleeding episodes usually occur from fragile telangiectatic lesions in the nasal or gastrointestinal mucosa. However, it has been proposed that impaired platelet function may reduce haemostasis and thus contribute to the pathogenesis of bleeding in HHT-patients [3–6]. Studies on this topic are few and conflicting results have been reported from impaired to increased platelet function [3–7]. Latest, Egido-Turrión et al. [8] described a common phenotype of impaired haemostasis in mouse models.

Bleeding episodes may be frequent, severe and often cause anaemia, but may also result in psychosocial morbidity, social isolation, and difficulty with routine daily activities [9]; therefore, knowledge of the causes for bleeding is relevant for better prevention of bleeding episodes and reduction in morbidity among HHT-patients.

This case–control study aimed to examine the platelet function in patients with hereditary haemorrhagic telangiectasia. The hypothesis was that patients with HHT and a history of moderate to severe bleeding events have impaired platelet function.

## Methods

### Study design, setting and participants

The study is a case–control study performed at the HHT-centre at Odense University Hospital, Denmark during April–October 2022. The inclusion criteria for patients were: (1) a genetically confirmed diagnose of either HHT1 or HHT2 also fulfilling clinical criteria of HHT [10], (2) age ≥ 18 years, (3) An epistaxis severity score (ESS) over 4 corresponding to moderate or severe nosebleeds [11]. Pregnant or breastfeeding patients and patients unable to pause medication affecting platelet function or the coagulation cascade for 5 days were excluded from the study. Patients had to pause intake of tranexamic acid for 24 h prior to blood sampling.

Healthy controls were recruited through advertisements at the hospital and the controls consisted primarily of healthcare professionals who were all ≥ 18 years of age and were not pregnant/breastfeeding, had no known systemic disease, and were not treated with any medication/supplements affecting the platelet function or the coagulation cascade.

Patients and healthy controls were invited to a physical visit where they were informed about the project and interviewed. All participants were offered at least 24 h to consider their participation before being enrolled in the study. Participation in the study included an interview and a blood sampling.

### Setting

All patients were recruited from the Danish National HHT-centre at Odense University Hospital. At the centre, patients were offered screening for and treatment of pulmonary arteriovenous malformations (PAVM). Screening for cerebral arteriovenous malformations (CAVM) was offered in any case of neurological symptom, and after close discussion with the patients. Screenings for hepatic arteriovenous malformations (HAVM) were performed in case of unexplained dyspnoea. Severity of epistaxis and GI-bleeding is evaluated in all patients. Anaemia and iron deficiency is diagnosed through blood test, and iron replacement therapy is started if indicated.

### Blood collection and laboratory analyses

Blood was sampled from a peripheral vein with a 21-gauge needle into BD Vacutainer tubes (Becton Dickinson, New Jersey, USA). Blood was kept at room temperature, and all analyses were performed within 3 h of collection.

Haematology parameters were measured on ethylenediaminetetraacetic acid (EDTA)-anticoagulated whole blood on Sysmex XN-9000 (Sysmex, Kobe, Japan). Fibrinogen (Dade Thrombin Reagent and Dade Owren’s veronal buffer) activated partial thromboplastin time (aPTT) (Dade actin FS Activated PTT Reagent), international normalized ratio (INR) (MRX Owren’s PT), and fibrin D-Dimer (Innovance D-dimer) were determined on citrate anticoagulated plasma with reagents from Siemens Healthcare GmbH (Elangen, Germany) on Sysmex CS-5100 (Sysmex, Kobe, Japan).

Concentrations of creatinine, C-reactive protein, potassium, sodium, alkaline phosphatase, albumin, bilirubin, alanine aminotransferase, cobalamin, iron, folic acid, gamma-glutamyl transferase, haptoglobin, transferrin and ferritin in plasma were measured from lithium heparin anticoagulated tubes on COBAS 8000 (Roche Diagnostics, Mannheim, Germany).

### Platelet function assessment

#### Platelet activation

Platelet activation capacity was measured with flow cytometry and comprised assessment of CD62p expression, CD63 expression, and PAC1 binding on platelet surface following platelet activation. It represents alpha granule and dense granule release and activation of the GPIIb/IIIa (fibrinogen) receptor, respectively [12]. In brief, tubes with 2.5 µL of citrate anticoagulated whole blood was incubated at room temperature for 15 min with 22 µL phosphate-buffered saline (PBS), 5.5 µL of antibody mix, and either 2.5 µL of platelet agonist for platelet activation or 2.5 µL ethylenediaminetetraacetic acid (EDTA) (10 mM) to prevent platelet activation in the negative sample.

The antibody mix consisted of: 14.6 µL phycoerythrin (PE)-conjugated glycoprotein (GP) Ib (CD42b, clone HIP1, dilution factor in final sample: 78), 1.8 µL allophycocyanin (APC)-conjugated P-selectin (CD62p, clone Psel.KO2.3, DF 325) (both eBioscience), 87.5 µL PE–cyanine 7 (Cy7) granulophysin (CD63, clone H5C6, DF 13), and 87.5 µL fluorescein isothiocyanate (FITC)-conjugated PAC-1 (DF 13) (both Becton Dickinson Bioscience, San Jose, CA, USA). In parallel, a tube serving as negative control sample contained 22 µL PBS and 5.5 µL of a mixture containing 4.2 µL PE-CD42b, 25 µL FITC-PAC-1, 25 µL PE–cyanine 7 (Cy7) granulophysin (CD63, clone H5C6, DF 13), and 1 µL of an anti-P-155 selectin matched APC-conjugated isotype control.

For platelet activation, three agonists were used: 1.1 μg/mL Collagen-related peptide (CRP) (Dr. Richard W. Farndale, University of Cambridge, UK), 10.0 μM thrombin receptor activating peptide (TRAP), or 12.8 μM adenosine diphosphate (ADP). One agonist was added to each respective tube.

After 15 min of incubation, the mixtures were fixated with 0.2% formalin in PBS. Results were acquired using the FACSCanto II (BD Bioscience, Becton, Dickinson and Company, NJ, USA) and results were processed using Kaluza software 1.3 (Beckman Coulter, California, USA). Gating on platelets was based on scatter patterns and GPIbα (CD42) expression. The platelet activation was presented as mean fluorescence intensities and the percentage of platelets positive for P-selectin, CD63, and the activated GPIIb/IIIa receptor compared to the negative sample.

#### Platelet aggregation

Platelet aggregometry analysis by flow cytometry was run as described in Vinholt et al. [12]. Before experiments were conducted, plasma from one AB-positive blood donor with no history of bleeding tendency was obtained. Donor plasma was made by double centrifugation of citrate-anticoagulated blood at 1000 g for 10 min, where the sediment was disposed after each centrifugation and stored at − 80°. The same batch of plasma was used for all experiments.

When testing platelet aggregation, platelet rich plasma (PRP) from patient or control was obtained by centrifugation at 200 g, 20 °C for 10 min and transferred to separate Nunc-cryo tubes (Thermo Fisher Scientific, Massachusetts, USA). To avoid platelet activation, acid-citrate-dextrose, and prostaglandin, E1 was added. From PRP, two tubes with platelet count of 144 * 10^9^ and 16 * 10^9^ were made by diluting with HEPES-buffer (134 mM NaCl, 2.9 mM KCL, 1 mM MgCl2, 5.6 mM glucose, and 20 mM 4-(2-hydroxyethyl)-1-piperazineethanesulfonic acid (HEPES), pH 7.4) and then labelled with calcein acetoxymethyl ester (AM) ultrapure grade (CAMU), and calcein (AM) violet 450 (CV450), respectively. The colours were prepared after the manufacturer’s instructions and diluted with phosphate buffered saline (PBS) to working stock concentration of 7.5 µM for CV450 and 1.25 µM for CAMU and incubated in the dark for 15 min at 37 °C.

Upon platelet aggregation testing, 35 µL of CAMU labelled platelets, 35 µL of CV450 labelled platelets are mixed with 70 µL of donor plasma (140 µL in total). Then, 3.5 µL platelet agonist was added per tube. The final concentration of agonist was 2.2 µg/mL for CRP, 244 µM for ADP, or 244 µM for TRAP, respectively. Furthermore, an unstimulated platelet mix (all components except the agonist) was analysed as negative control in parallel. The tubes were shaken for 5 min, 1000 rpm, 37 °C in an Eppendorf thermomixer comfort (Eppendorf, Hamburg, Germany) and 10 µL of the mixture was then transferred in to 400 µL fixation buffer (Formalin (0.2% in PBS)). Results were obtained as described in Vinholt et al. [12], using FACS DIVA software (Becton Dickinson). Platelet aggregation was reported as the percentage of calcein-AM double-positive events (positive for both CV450 and CAMU) out of all CV450 positive events.

#### Platelet function analyzer 200

Platelet Function Analyzer 200 (PFA-200) measures the haemostasis (platelet adhesion and aggregation) in a realistic haemodynamic environment, since the setting imitates a blood vessel. In brief, 800 µL of citrate-anticoagulated whole blood was added to a disposable cartridge (ADP/collagen) and aspirated through a capillary in the instrument cartridge where an ADP and collagen coated membrane activates the primary haemostasis. The tests were conducted according to the manufacturer's instructions for the PFA-200 (Siemens Healthcare GmbH, Erlangen, Germany). Results were reported as closure time (CT) in seconds.

#### Thromboelastometry

The overall haemostatic function was assessed by rotational thromboelastometry (ROTEM® *sigma,* Tem Innovations gmbH, Munich, Germany), which graphically displays the dynamic development of a blood clot over time. Citrated blood samples (2.7 mL) and reagents from the manufacturer were used for thromboelastometry, and the following assays were evaluated: EXTEM (evaluation of the extrinsic coagulation pathway), INTEM (evaluation of the intrinsic coagulation pathway), FIBTEM (isolated evaluation of fibrinogen polymerization), and APTEM (an assay containing aprotinin, which detects hyperfibrinolysis). The results contained information regarding clotting time (CT), maximum clot firmness (MCF) and maximum lysis of the clot (ML). Rotational thromboelastometry analyses were conducted for HHT-patients only.

#### Statistics

Sample size calculation was based on platelet function evaluated as percentage of CD62p positive platelets after stimulation with ADP, which previously has been shown to be 97.1% ± 1.4 in healthy individuals [13]. Based on a power of 90% and alpha of 0.05, we would be able to show a 10% lower platelet function in HHT-patients compared to healthy controls if 10 persons in each group were included. As we expected a larger variation in the results from HHT-patients than for healthy individuals, we included at least 20 participants per group.

We evaluated data distribution by Shapiro–Wilk test and inspection of histograms. We presented categorical variables as numbers and percentages. We described continuous variables as mean and standard deviation (SD) for normally distributed data and median and interquartile ranges (IQRs) for non-normally distributed data. When comparing the two groups (HHT-patients versus healthy controls and subgroup comparisons of HHT patients) based on continuous variables, we used t-test for normally distributed data and Wilcoxon Rank-sum test for non-normally distributed data and Fisher’s exact test for categorical values.

In secondary analyses, we did logistic regression with adjustments for age and gender to examine the relationship between HHT and platelet function. Then we examined if platelet function correlated with age in the HHT group and if platelet function differed based on sex in the HHT group. We also investigated whether platelet function was reduced and other laboratory results differed in patients with higher (> 6) versus lower ESS-score (≤ 6), Furthermore, Pearson correlation analysis between ESS-score as continuous variable and platelet function parameters was done to explore if an inverse linear relationship exists between bleeding phenotype and platelet function. Overall, *p*-values were two-tailed and considered statistically significant when *p* < 0.05. Statistical analyses were performed using Microsoft Excel (Microsoft 365 MSO, version 2207), Stata software package (Stata 13.1; StataCorp, College Station, TX, USA) and GraphPad Prism 9.4.1 (GraphPad Software, La Jolla, CA, USA)**.**

## Results

### Background variables

In total, 22 HHT-patients and 20 healthy controls were enrolled in this study. Characteristics of participants are presented in Table 1. There was no difference in gender distribution between groups, *p* = 0.76, but controls were younger than HHT-patients, median 54 years (IQR 40–62) versus 61 years (IQR 50–72), *p* = 0.049. Twelve patients had HHT-type 1(ENG-gene-mutation) and 10 patients had HHT-type 2 (ACVRL1-gene-mutation). Of the HHT-patients, six were active smokers, six had hypertension, three had diabetes mellitus II and four had a systemic inflammatory disease (allergy and arthritis). Twelve patients (7 men and 5 women) were treated with iron (infusion or tablets), one with SSRI, six with tranexamic acid (all paused for 24 h before enrolment) and three with platelet inhibitors. The medications the participants consumed was acetylsalicylic acid as analgesics (n = 1) and as thrombosis prophylaxis (n = 2), all paused for 5 days before enrolment. The mean ESS-score among HHT-patients was 6.0 (IQR 5–6.5). There was no correlation between ESS scores and age in this group (r = 0.25, *p* = 0.3). When comparing results based on sex, we found no difference in ESS score based on sex (p = 0.97). PAVM were part of the clinical picture in 9 patients, CAVM in 1, and HAVM in 5. As the patients had moderate to severe bleeding, some of them were heavy bleeders. Efforts were made to perform the blood test before treatment. One patient had received 8 Units of blood within 4 weeks before blood test and that patient had also had electrocoagulation of the nasal mucosa in local anaesthesia. Another patient had received 2 units of blood in a 4-week period before blood test, this patient also had electrocoagulation of the nasal mucosa in that case in general anaesthesia. One patient had experienced nasal bleeding and had electrocoagulation of the nasal mucosa in local anaesthesia.

**Table 1:** Clinical characteristics of 22 HHT-patients and 20 healthy controls

	Controls (n = 20)	HHT-patients (n = 22)
Female sex, n (%)	11 (55%)	10 (45%)
Years, median age (IQR)	54 (40–62)	61 (50–72)
Smoking, n (%)	1 (5%)	5 (22.7%)
*Co-morbidity*		
Hypertension	0 (0%)	6 (27%)
Diabetes mellitus II	0 (0%)	3 (13.6%)
Systemic inflammatory disease	0 (0%)	4 (18%)
*Medical treatment*		
Iron treatment (pill/infusion)	0	9 (40%)/3 (13.3%)
SSRI	0	1 (4.5%)
NSAID	0	0
Tranexamic acid	0	6 (26.6%)
Platelet inhibitors	0	3 (13.3%)
Avastin	0	0
Iron replacement therapy	0	12
Blood transfusions within the last 4 weeks	0	2
Electrocoagulation of nasal mucosa within last 4 weeks	0	3
*HHT-type, n (%)*		
Type 1		12 (54.5%)
Type 2		10 (45.5%)
Epistaxis severity score (ESS), median (IQR)		6 (5–6.5)
*Arteriovenous malformations*		
PAVM		9 (40%) 5 needed further embolization
CAVM		1 (5%)
HAVM		5 (23%)

*IQR* interquartile range, *PAVM* pulmonary arteriovenous malformations, *HAVM* hepatic arteriovenous malformations, *CAVM* cerebral arteriovenous malformations (CAVM), *NSAID* non-steroid anti-inflammatory drug, *SSRI* selective serotonin reuptake inhibitors, *Y* years. Data are mean and standard deviation, or median and interquartile range. Tranexamic acid was paused for 24 h, and platelet inhibitors were paused for 5 days. All patients were offered screening for PAVM, this was performed in all 22, 9 (40%) had PAVM

### Baseline laboratory analyses

While standard laboratory analyses showed that HHT-patients had a higher platelet count compared to the control group, no significant difference in mean platelet volume and immature platelet fraction between the two groups were found. In relation to the control group, the HHT-patients had lower levels of bilirubin, albumin, and a higher level of gamma-glutamyl transferase. The patients also had lower haemoglobin, iron, ferritin, higher transferrin and reticulocyte count than controls. Among HHT patients, 14 (64%) had anaemia, while no controls had anaemia. The results of all other standard analyses were without significant difference between HHT-patients and controls (*p* > 0.05), see Table 2. In secondary analyses, we compared the HHT patients’ results based on ESS score (> 6 vs.  ≤ 6). There was no difference in laboratory variables, except for creatinine. When comparing results in patients with HHT based on gender, we found a higher creatinine among men, mean 91 µmol/L (SD 26) versus 64 µmol/L (SD 6.8), *p* = 0.004. No difference was observed for other laboratory variables.

**Table 2:** Laboratory data of 22 HHT-patients and 20 healthy controls

	Laboratory reference interval	Controls (n = 20)	HHT-patients (n = 22)	*p*-values
Platelet count, 10^9^/L	F: 145–350; M:165–400	254 (IQR 230–287)	288 (IQR 250–381)	0.046
Mean platelet volume, fL (SD)	6–11	9.5 (IQR 9.1–10.4)	9.6 (IQR 9.2–10.0)	0.93
Immature platelet fraction, %	0.011–0.061	0.025 (IQR 0.016–0.041)	0.027 (IQR 0.018–0.035)	0.90
Fibrinogen µmol/L	5.5–11.5	8.7 (± 1.6)	9.9 (± 1.6)	0.27
D-dimer, mg/L FEU	0-55y: < 0.5; 55–65y: ≤ 0.6; 65–75y: ≤ 0.7; 75–85y: ≤ 0.8 85–95y: ≤ 0.9	0.20 (IQR 0.19–0.34)	0.37 (IQR 0.19–0.53)	0.91
INR (factor II + VII + X)	0.9–1.1	1.0 (IQR 1–1.01)	1.0 (IQR 1–1.05)	0.72
APTT, seconds	27–40	24 (± 1)	24 (± 2)	0.54
Folate, nmol/L	> 7	13.5 (IQR 11–20)	15.0 (IQR 9–29)	0.71
Haemoglobin, mmol/L	F: 7.3–9.5, M: 8.3–10.5	8.8 (± 0.8)	7.2 (± 1.5)	0.0001
MCV, fL	85–100	88.5 (IQR 85–91)	86 (IQR 81–93)	0.38
Reticulocytes, 10^9^/L	F: 29–103, M:32–112	65 (IQR 51–80)	84 (IQR 73–118)	0.008
MCHC, mmol/L	19.7–22.2	21.2 (IQR 21.1–21.6)	20.3 (IQR 19.4–21.0)	0.33
Iron, µmol/L	9–34	20 (IQR 14–24.5)	8 (IQR 5–12.5)	0.0048
Ferritin, µg/L	F: 15–180, M: 15–560	73.5 (IQR 47–123)	37.5 (IQR 22–68)	0.015
Transferrin, µmol/L	25–45	33 (IQR 31–35)	37 (IQR 32–40)	0.034
Transferrin saturation, %	F: 0.1–0.5, M:0.15–0.57	0.29 (IQR 0.2–0.37)	0.11 IQR (0.05–0.18)	0.19
White blood cell count, 10^9^/L	3.5–8.8	5.8 (± 1.1)	5.8 (± 1.8)	0.89
Creatinine, µmol/L	F: 45–90, M: 60–105	69 (IQR 64–78)	72 (IQR 66–83)	0.44
Albumin, g/L	15–40 y: 36–50, 40–70 y: 36–48, ≥ 70 y: 34–45	48 (IQR 46–50)	45 (IQR 43–47)	0.002
Alanine aminotransferase, U/L	F: 10–45, M: 10–70	24 (IQR 19–31)	22 (IQR 19–28)	0.70
Gamma-glutamyl transferase, U/L	F: 18-40y:10–45, > 40y: 10–75, M: < 40: 10–80, > 40: 15–115	16.5 (IQR 13–20.5)	29.0 (IQR 18–56)	0.038
Alkaline phosphatase, U/L	35–105	65 (IQR 56–71)	83 (IQR 65–90)	0.053
Bilirubin, µmol/L	5–25	8 (IQR 7.8–11)	6 (IQR 4–9.3)	0.0035
Potassium, mmol/L	3.5–4.4	3.9 (± 0.2)	4.0 (± 0.35)	0.50
Sodium, mmol/L	137–145	139.6 (± 1.6)	139.1 (± 2.5)	0.48
Calcium ionized, mmol/L	1.18–1.32	1.3 (± 0.03)	1.2 (± 0.05)	0.68
Lactate dehydrogenase, U/L	< 70y: 105–205, > 70y: 115–255	189 (± 24)	192 (± 32)	0.74
Cobalamin, pmol/L	200–600	288 (IQR 253–317)	281 (IQR 240–424)	0.92

*IQR* interquartile range, *F* female, *M* male, *Y* years, *fL* femtoliter, *INR* international normalized ratio, *aPTT* activated partial thromboplastin time. Data are mean and standard deviation, or median and interquartile range. T-test and Wilcoxon Rank-sum test generated the *p*-values

### Platelet function

**Fig. 1:**
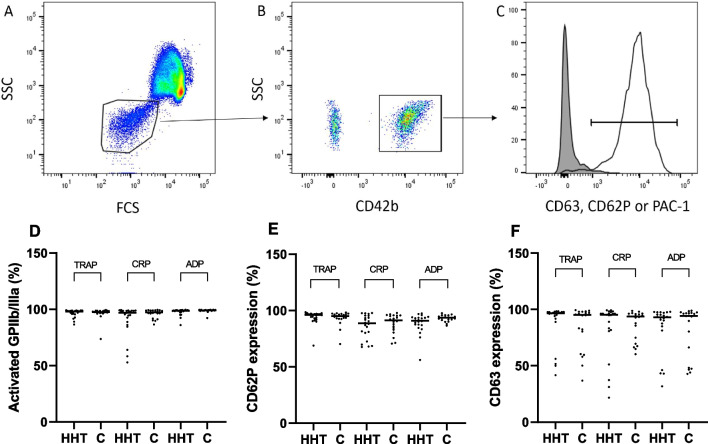
Platelet function results in patients with hereditary haemorrhagic telangiectasia (n = 22) and healthy controls (n = 20). **A**–**C** illustrates the gating strategy applied for CD62P expression. **A** The dot plot demonstrates the discrimination of platelets from red blood cells (RBCs) based on size and content using forward scatter and side scatter characteristics, followed by the ubiquitously expressed platelet membrane marker (CD42b) (**B**). The baseline expression of platelet activation markers was defined using a non-stimulated EDTA-sample (dark grey population) (**C**). Platelet function results are reported as dots, and the horizontal line is median for the group. D, E and F display the platelet activation in terms of the percentage of platelets positive for activated GPIIb/IIIa, CD62P, CD63, and after stimulation with agonist in comparison to the negative control sample. Results are analysed by flow cytometry after gating on platelets [14]. *ADP* adenosine diphosphate, *TRAP* thrombin receptor-activator peptide, *CRP* collagen related peptide

**Fig. 2:**
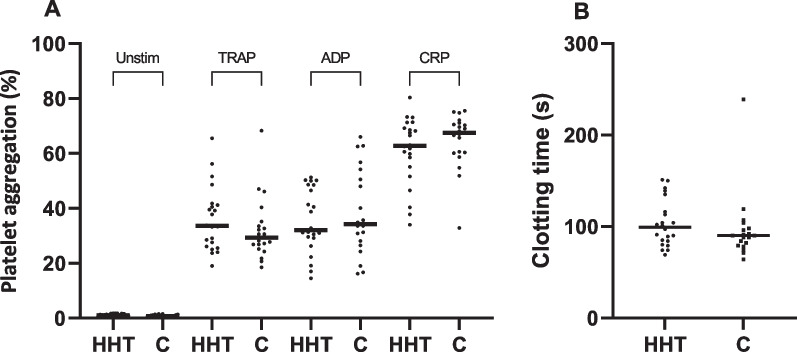
Platelet function results in patients with hereditary haemorrhagic telangiectasia (n = 22) and healthy controls (n = 20). Results are reported as dots, and the horizontal line is median for the group. A displays the platelet aggregation assessed by flow cytometry performed in accordance with Vinholt et al. [12]. B reflects the platelet function as clotting time assessed by Platelet Function Analyzer 200. *ADP* adenosine diphosphate, *TRAP* thrombin receptor-activator peptide, *CRP* collagen related peptide

We found no significant difference in platelet activation capacity in terms of the percentage of platelets positive for P-selectin, CD63 or activated GPIIb/IIIa for any used agonist when comparing results from HHT-patients and the control group. Neither did we find a difference in mean fluorescence intensities, data not shown; Details are shown in Fig. 1. Accordingly, we used the flow cytometric platelet aggregation analysis and found no difference in platelet aggregation response in HHT-patients compared with the control group for any used agonist (ADP, TRAP and CRP), see Fig. 2.

When evaluating the global haemostatic response with PFA-200, we congruently found no significant difference in closure time in the two groups. HHT-patients median CT on 99 (84–115) versus healthy controls median CT on 90 (80–97), *p* = 0.2, see Fig. 2.

The global coagulation response with ROTEM was only addressed for HHT-patients. None of the patients had values outside the laboratory reference range for CT and ML. No patients had values below the reference range regarding MCF. However, some patients exceeded this reference range. Overall, seven, three and five patients had MCF values exceeding the reference range in EXTEM, INTEM and APTEM respectively. See results in Table 3.

**Table 3:** ROTEM-data from 22 HHT-patients

	Fibtem			Extem			Intem			Aptem		
	CT	MCF	ML	CT	MCF	ML	CT	MCF	ML	CT	MCF	ML
Reference range	38–79	9–25	0–15	38–79	50–72	0–15	100–240	50–72	0–15	38–79	50–72	0–15
Mean (± SD)	58.5 (± 5.5)	15.5 (3.9)	0.0 (± 0.0)	58 (± 5.4)	70 (± 4.5)	1.5 (± 2.0)	181.5 (± 17.5)	67.5 (± 5.3)	2.5 (± 2.5)	55.5 (± 5.7)	68.5 (± 4.9)	1.0 (± 2.1)
N above reference range	0	0	0	0	7	0	1	3	0	0	5	0

*CT* clotting time in seconds, *MCF* maximum clot firmness in mm, *ML* maximum lysis in %. No values were below the reference range

In secondary analyses, we did logistic regression with adjustments for age and gender. Platelet function results were not significantly different in HHT groups after adjustments, *p* > 0.05. When comparing results based on gender in the HHT group, there was no difference in platelet function, except for a higher percentage of platelets positive for PAC1 binding after stimulation in women with CRP, mean 97% (± 2.4) in women versus 85% (± 1.7) in men, *p* = 0.04. Men also had higher MPV and IPF than women. Mean MPV was 10.1 fL (± 9.7) in men versus 9.2 fL (± 0.6) in women, *p* = 0.01 and IPF was 0.05% (± 0.04) versus 0.02% (± 0.01) in women, *p* = 0.04. Correlation analyses between age and platelet function results in the HHT group showed no statistically significant associations, *p* > 0.05. Evaluation of data from HHT patients grouped by ESS-score, showed no difference in platelet function between patients with high (> 6) and low ESS-score (≤ 6). Further correlation analysis showed no correlation between ESS-score and platelet function tests.

## Discussion

We investigated 22 HHT-patients having moderate to severe nosebleeds and 20 healthy controls, and the main finding of this study was that platelet parameters were not impaired among HHT-patients who had nosebleeds (ESS ≥ 4).

This study included HHT-patients with a significant bleeding history. Also, there was a high frequency of iron deficiency anaemia despite iron replacement in the group, but no gender-based differences in bleeding severity based on ESS or the degree of anaemia. In the Danish HHT center, we routinely measure haemoglobin and iron levels, and recommend oral or intravenous iron therapy, as necessary. Further, efforts are put into informing and assisting patients through leaflets and an app. Despite this, iron deficiency remains a persistent concern that requires further research. The present study evaluates if platelet function impairment is involved in HHT and thus, should be considered in the care of these patients.

Within the research field of HHT, it has been suspected that impaired platelet function could contribute to the bleeding diathesis of HHT-patients. Previous studies on platelet function in HHT-patients are few [3–6, 15] and mostly case-reports with divergent results. Some report decreased platelet function [3–5], a single study report enhanced platelet function [15] and normal platelet function in patients with HHT is reported in a single study [6]. Among these studies, one used an obsolete method [5] and another did not describe the method used [3]. Amongst the remaining three, one is a case report of a patient with HHT who had decreased ADP-, epinephrine- and collagen-induced platelet aggregation measured by light transmission aggregometry [4]. Recent case–control studies on seven patients in each study showed normal platelet aggregation (light transmission aggregometry) for ADP, epinephrine, collagen, arachidonic acid, and ristocetin [6] but enhanced platelet aggregation for serotonin [15] compared with healthy controls.

In the present study, we provided a thorough investigation of platelet activation and aggregation and found no impairment of platelet function in patients with HHT. Further, we evaluated global evaluation of primary haemostasis using PFA-200 and found no difference between patients and controls. This test imitates global response to vessel damage, as blood is drawn at high pressure through an aperture coated with platelet agonists, leading to adhesion, activation, and aggregation of platelets. Moreover, we observed no patients with reduced response through secondary haemostasis using ROTEM where platelets contribution to clot strength is addressed. The ROTEM analyses showed no signs of hyperfibrinolysis (all patients had normal ML), but some patients had elevated MCF, that may indicate increased clot firmness. However, these values only slightly exceeded the reference range. Overall, patient stratification showed difference in platelet function when examining function within the patient group. Thus, there was no correlation between platelet function and bleeding score.

Except for platelet aggregation, all analyses were performed on whole blood, which limits any potential bias introduced by in vitro handling of platelets. Our study contributes to existing knowledge with a comprehensive display of platelet function in a large group of HHT-patients and was based on a calculated sample size. The inclusion criterion was significant bleeding history, which ensures that the findings probably are applicable for HHT-patients with minor bleeding symptoms as well. We took precautions for this study, as patients had to pause their intake of tranexamic acid and medication that affect platelet function for 24 h and 5 days, respectively.

The current study focused on haemostatic markers of platelets based on the controversy in existing evidence, and no evidence exist regarding the role of platelets’ non-haemostatic functions in HHT, which is a relevant area for future studies. [16] However, it shall be emphasized that other so-called ‘non-haemostatic’ properties of platelets, may be relevant for the prevention of bleeding including receptors relevant for platelet adhesion during inflammation, e.g., platelet surface expression the collagen receptor, GPVI and angiogenetic factors from platelets that could be directly involved in the pathogenesis of HHT. Furthermore, our methods did not reflect the platelet function in relation to platelet-endothelial interaction in vivo. In this regard von Willebrand factor levels would be of interest due to the importance in platelet adhesion to the endothelium and vasculature. Egido-Turrión et al. [8] recently has shown that HHT-1 mice have affected thrombus stabilization and HHT-2 mice have an upregulation in the fibrinolytic system. Comorbidities and medication often have the potential to impair platelet function. In this study, patients had comorbidities and were undergoing medical treatment, which could reasonably be expected to influence their platelet function negatively. Despite this, our analysis did not reveal any significant difference in platelet function between patient groups. Nevertheless, the study is relevant for addressing the role of platelet haemostatic properties in HHT bleeding that has been raised in the literature.

## Conclusion

Overall, there were no significant differences in platelet function between HHT-patients and healthy controls. Reduced platelet function is therefore unlikely to contribute to the many and long bleeding episodes that these patients suffer from. We propose that further studies should focus on whether patients with HHT have hypercoagulability.

## Data Availability

The data that support the findings of this study are not openly available due to reasons of sensitivity and are available from the corresponding author upon reasonable request. Data are in an online controlled data storage, REDcap at OPEN, Patient Data Explorative Network, J.B. Winsløws Vej 9A, third floor, 5000 Odense C, Denmark. Available until February 2024.
